# Potential domino effect of the 2023 Kahramanmaraş earthquake on the centuries-long seismic quiescence of the Dead Sea fault: inferences from the North Anatolian fault

**DOI:** 10.1038/s41598-024-65906-4

**Published:** 2024-07-04

**Authors:** Erhan Altunel, Özgür Kozacı, Cengiz Yıldırım, Reda Mohamed Sbeinati, Mustapha Meghraoui

**Affiliations:** 1 Jeoloji Mühendisliği Bölümü, Eskişehir Osmangazi Üniversitesi, Eskişehir, Turkey; 2 Geosciences, Pacific Gas and Electric, Oakland, CA USA; 3 Eurasia Institute of Earth Sciences, Istanbul Technical University, Sarıyer, 34460 İstanbul, Türkiye; 4 SAEC, Damascus, Syria; 5 Institut Terre et Environnement, UMR 7063, University of Strasbourg, Strasbourg, France

**Keywords:** Natural hazards, Solid Earth sciences

## Abstract

Field observations conducted immediately following the February 6, 2023, Mw 7.8 Kahramanmaraş earthquake documented the southern surface rupture termination in the Amik Basin. The termination occurred in an en-echelon pattern, extending across the 3.5 km width of the approximately 10-km-wide stepover. This extension reached towards the northern tip of the Hacıpaşa Fault, which constitutes the main northern segment of the Dead Sea Fault Zone (DSFZ). Archaeoseismologic and paleoseismologic data show that the approximately 800-km-long DSFZ has been seismically quiet for more than 600 years in the north and 900 years in the south. A similar fault connection geometry at the western end of the 1939 Ms 7.9 Erzincan earthquake in the easternmost part of the North Anatolian Fault Zone and the subsequently triggered successive large magnitude earthquakes migrating westward within a few decades highlights an increased seismic hazard for the entire DSFZ. This heightened seismic hazard potential along the DSFZ, combined with historical population centers experiencing wars and migrations, puts millions of people at an unparalleled risk.

## Introduction

**Figure 1:**
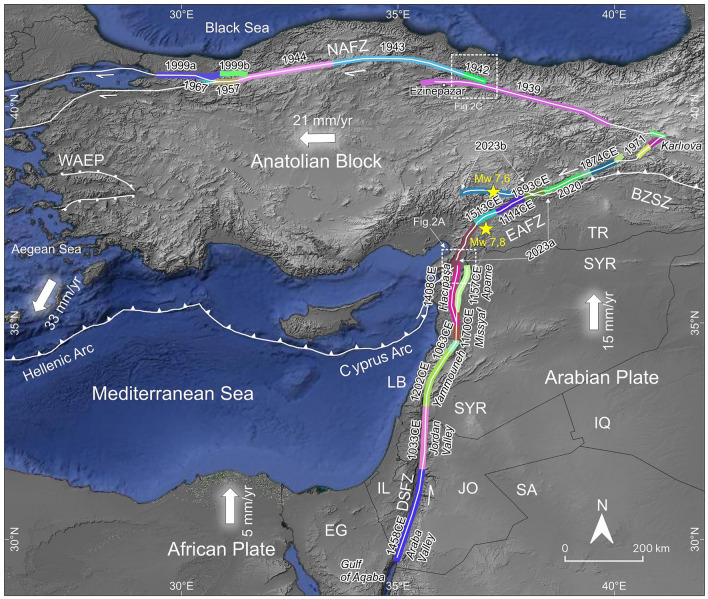
Major active faults in the eastern Mediterranean region and large magnitude historical earthquake ruptures along the DSFZ, EAFZ and NAFZ. NAFZ: North Anatolian Fault Zone, EAFZ: East Anatolian Fault Zone, DSFZ: Dead Sea Fault Zone, BZSZ: Bitlis-Zagros Sture Zone, WAEP: Western Anatolian Extensional Province. Large white arrows with numbers indicate plate motion direction and rate. Year on different color of the fault zones is the most recent surface rupturing earthquake attributed to that section. TR: Türkiye, SY: Syria, IQ: Iraq, LB: Lebanon, IL: Israel, EG: Egypt, JO: Jordan, SA: Saudi Arabia. (Redrawn using data from^4,6–9^, the figure was created in QGIS-LTR 3.28.15 Firenze, an open-source geographical information system software https://www.qgis.org/en/site/forusers/download.html. The background DEM is generated from SRTM, resolution 3-ARC (90 m), https://earthexplorer.usgs.gov).

**Figure 2:**
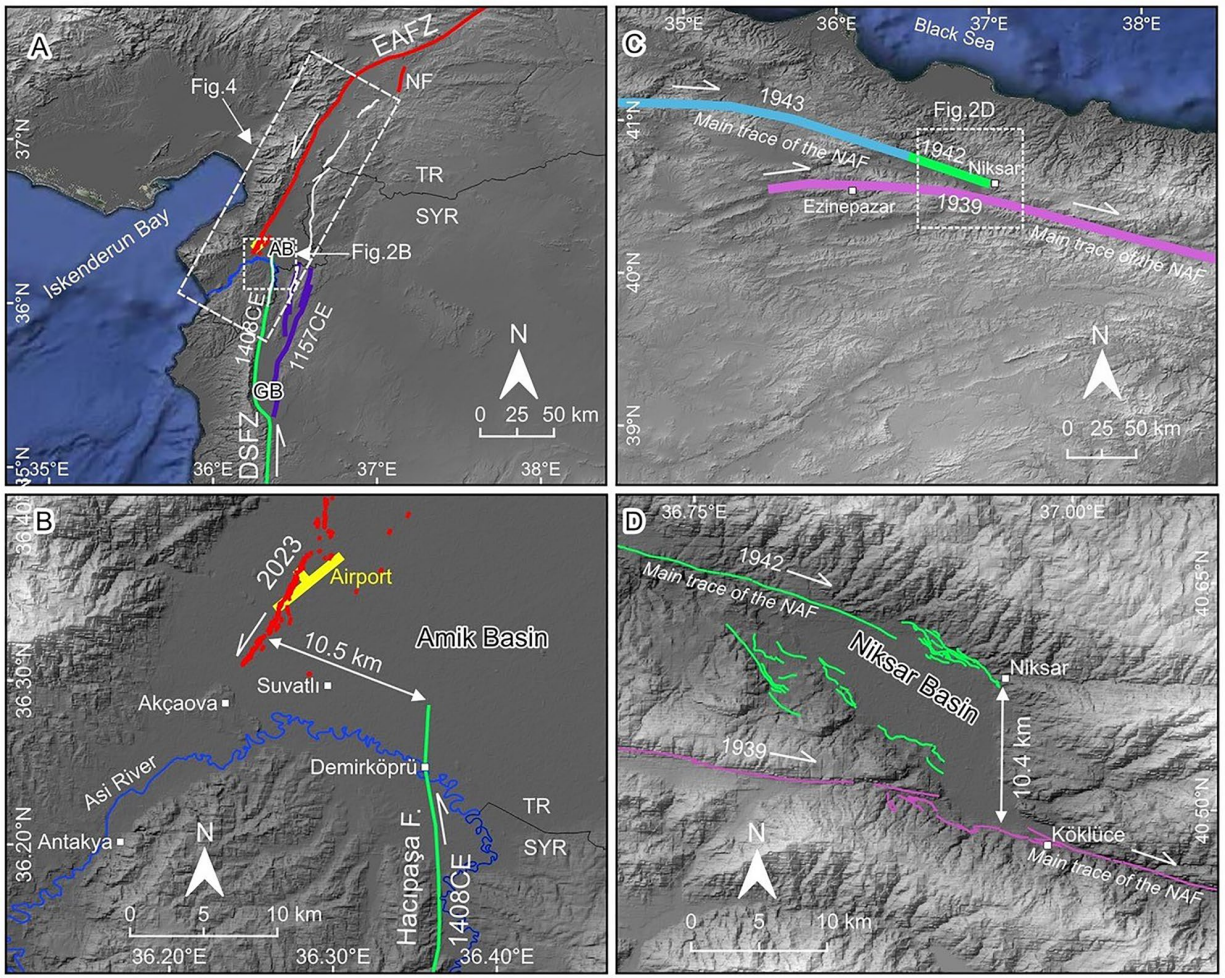
(**A**) Faults in the Eastern Mediterranean region, solid red lines are the surface rupture of the February 6, 2023, Kahramanmaraş earthquake. (White lines are from the active fault map of Türkiye^8^, faults in Syria are from^9^). AB: Amik Basin, GB: Ghab Basin. (**B**). Detailed map of the southernmost end of the 2023 surface rupture in the Amik Basin. Rupture steps over ~ 3.5 km to the southeast and dies immediately west of the Suvatlı village. (**C**) Map of main North Anatolian fault segments around the Niksar Basin. Note that the 1939 earthquake surface rupture did not propagate westward along the main trace of the NAFZ. (Faults are from^4^). (**D**) The rectangle with a dashed line in panel C indicates the boundary of this panel. Detailed map of the 1939 and 1942 earthquake ruptures around the Niksar Basin (faults are from^8^). (Figures were created in QGIS-LTR 3.28.15 Firenze, an open-source geographical information system software https://www.qgis.org/en/site/forusers/download.html. The background DEM is generated from SRTM, resolution 3-ARC (90 m), https://earthexplorer.usgs.gov).

The East Anatolian Fault Zone (EAFZ) is one of the major active strike-slip fault zones of the Eastern Mediterranean region, accommodating deformation between the Anatolian Block and Arabian Plate (Fig. 1). On February 6, 2023, the Mw 7.8 Kahramanmaraş earthquake initiated on the Narlı Fault (Fig. 2A) and reactivated the main trace of the EAFZ, generating an ~ 375 km long surface rupture from northeast of Çelikhan in the north to Amik Basin in the south^1^. Detailed mapping of the southernmost extent of the Kahramanmaraş earthquake rupture revealed an ~ 10-km-wide releasing stepover between the southern end of the rupture and the northernmost segment of the Dead Sea Fault Zone (DSFZ) (Figs. 1, 2B)^1^.

**Figure 3:**
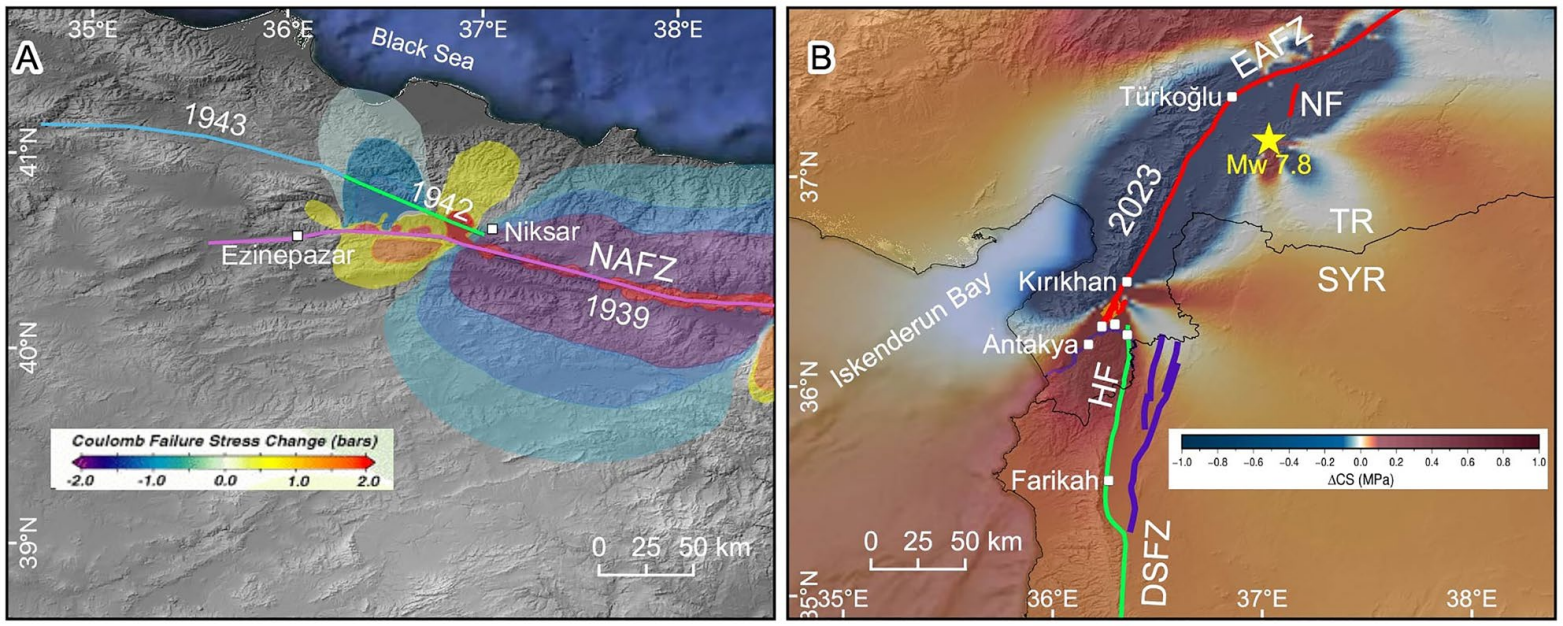
(**A**) Coulomb static stress change model (from^2^) indicates stress increase at the western end of the 1939 rupture across the 10 km wide Niksar Basin where three years later, Ms 7.0 1942 earthquake was triggered. NAFZ: North Anatolian Fault Zone. (**B**) Coulomb static stress change model (from^5^) indicates stress increase on the Hacıpaşa Fault (HF) in the northern tip of the Dead Sea Fault Zone (DSFZ). EAFZ: East Anatolian Fault Zone, NF: Narlı Fault. Faults in Syria are from^9^. (Figures were created in QGIS-LTR 3.28.15 Firenze, an open-source geographical information system software https://www.qgis.org/en/site/forusers/download.html. The background DEM is generated from SRTM, resolution 3-ARC (90 m), https://earthexplorer.usgs.gov).

An intriguing earthquake behaviour along major active fault zones is the domino-style triggering of large-magnitude earthquakes on neighbouring segments as a result of Coulomb stress transfer^2,3^. The 20th-century earthquake sequence along the North Anatolian Fault Zone (NAFZ) exemplifies this phenomenon. On December 27, 1939, the Erzincan earthquake (Ms 7.9) initiated in the eastern part of the NAFZ and ruptured approximately 360 km-long section (Fig. 1)^4^. The surface rupture of the 1939 earthquake followed the main trace of the NAFZ east of Niksar Basin, which is an ~ 10-km-wide releasing stepover on the NAFZ (Figs. 1, 2C). The surface rupture extended westward along the southern margin of the Niksar Basin because it could not cross the ~ 10 km wide releasing stepover (Fig. 2D). However, the 1939 event loaded significant stress on the segment bounding northern margin of the Niksar Basin (Fig. 3A) and three years later initiated the 20th-century earthquake sequence on the NAFZ from east to west^2^, starting with 1942 (Ms 7.0), and followed by 1943 (Ms 7.6), 1944 (Ms 7.4), 1957 (Ms 7.1), 1967 (Ms 6.8), 1999a İzmit (Mw 7.4) and 1999b Düzce (Mw 7.2) earthquakes (Fig. 1). Examination of geological parameters of the 1939 Erzincan and 2023 Kahramanmaraş earthquake terminations show a noteworthy similarity between these two events from a structural geology and seismic hazard perspective with potentially significant consequences. On both strike-slip faults, the width of the step-overs (both of them ~ 10 km, Fig. 2B,D) magnitudes (Ms 7.9 vs Mw 7.8) and the lengths of the surface ruptures (~ 360 km vs ~ 375 km) are very similar. As in the 1939 event (Fig. 3A)^2^, the Coulomb stress change analysis of the February 6, 2023, earthquake^5^ reveals stress loading on the neighbouring Hacıpaşa Fault, the northernmost segment of the DSFZ (Fig. 3B). In addition, a centuries-long seismic quiescence along the DSFZ starting from the ~ 10 km wide stepover in the southernmost termination of the Kahramanmaraş earthquake surface rupture further elevates the seismic hazard potential along the entirety of the DSFZ. In this paper, we present the structural and seismic activity similarities between the twentieth century NAFZ earthquake sequence and the connection between EAFZ and DSFZ and draw attention to the heightened seismic hazard potential between the southern termination of the Kahramanmaraş earthquake surface rupture in the north and the Gulf of Aqaba in the south, a socio-economically fragile region of the world.

### Tectonic setting and seismicity

**Figure 4:**
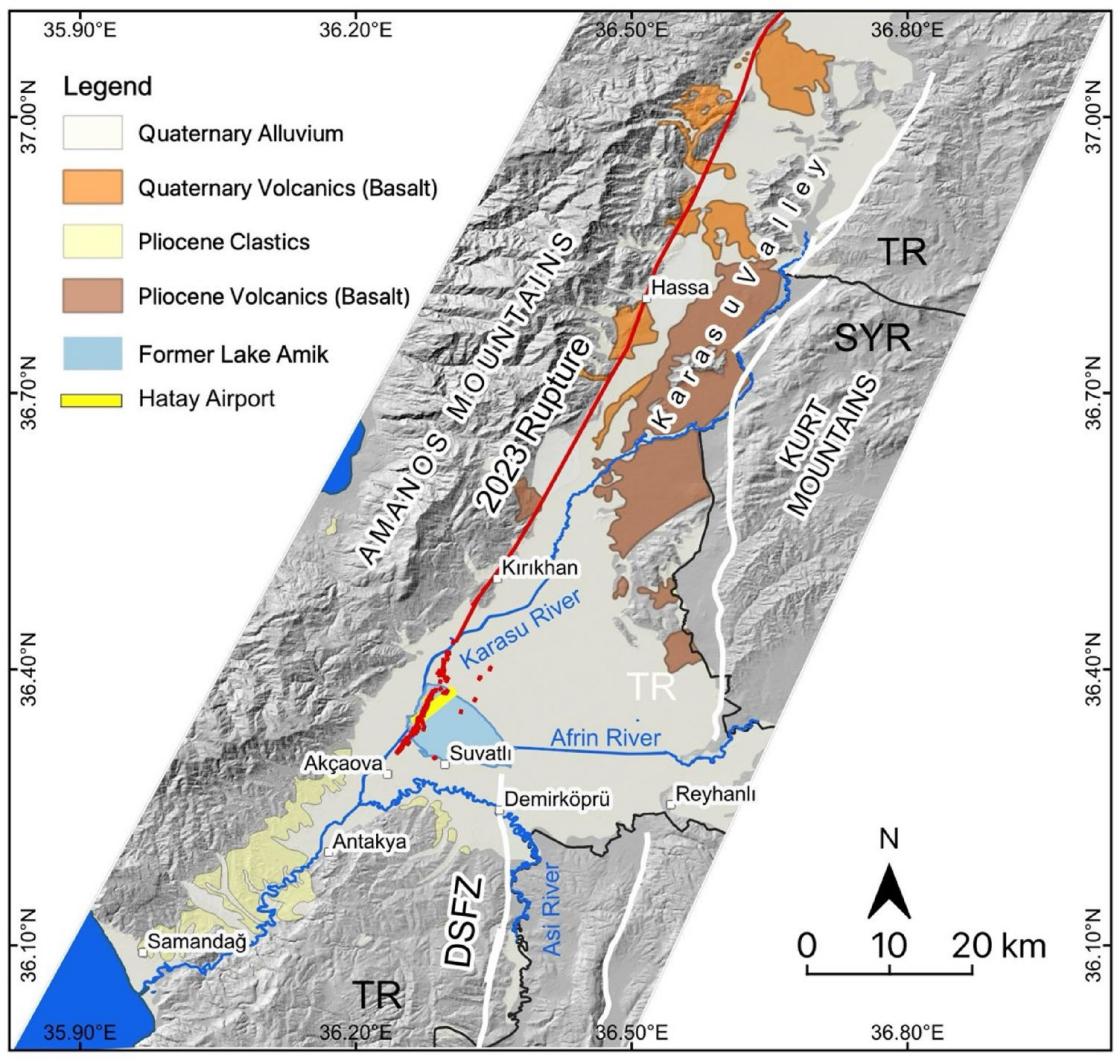
Simplified geological and active fault map of the Amik Basin area. Geological units are from^13,17^ and active faults (white lines) in east of Karasu Valley are from^8^. (The figure was created in QGIS-LTR 3.28.15 Firenze, an open-source geographical information system software https://www.qgis.org/en/site/forusers/download.html. The background DEM is generated from SRTM, resolution 1-ARC (30 m), https://earthexplorer.usgs.gov).

Principal tectonic structures governing the tectonics of the Eastern Mediterranean region in the Eurasian-Arabian collision zone are the NAFZ to the north and the EAFZ and DSFZ to the south (Fig. 1). The NAFZ is a right-lateral strike-slip fault zone extending approximately E-W between the northern Aegean Sea in the west and Karlıova in the east. Almost the entire NAFZ ruptured with M > 7 westward migrating earthquakes during the twentieth century. The left lateral EAFZ starts from Karlıova, intersecting the NAFZ and extends southwestward towards the İskenderun Bay (Fig. 1). The EAFZ connects with the DSFZ through the Karasu Valley (Fig. 2A). The left lateral DSFZ extends from the Gulf of Aqaba in south to the EAFZ in north (Fig. 1). The northernmost continuation of the DSFZ splays into branches in southern Türkiye (Fig. 4) and the slip is partitioned. However, geological, geomorphological, palaeoseismological and archaeoseismological studies^10–14^ suggest that the main branch of the DSFZ (Hacıpaşa Fault) enters the Amik Basin in N-S direction, steps over to the west in the basin and extends in NE-SW direction along the western side of the Karasu Valley (Fig. 4). The significant amount of slip is taken by the Hacıpaşa Fault and further north by the fault that extends along the western side of the Karasu Valley. The slip on the DSFZ is transferred northward via the faults mapped on both sides of the Karasu Valley and numerous extensional cracks that develop on the valley floor, and as Fig. 2A shows the junction between the EAFZ and DSFZ is near Türkoğlu^15,16^.

Several moderate to large magnitude earthquakes occurred on the EAFZ during the nineteenth century^6^ and instrumental period. The DSFZ is a left lateral fault zone extending approximately northward from the Gulf of Aqaba towards the Amik Basin (Fig. 1). The main segments of the DSFZ that start from the Amik Basin are Hacıpaşa, Missyaf, Yammouneh, Jordan Valley and Araba Valley segments from north to south, respectively. The DSFZ usually has a simple geometry in the south, but it bifurcates into multiple branches towards the north (Fig. 2A). The main northernmost branch is the Hacıpaşa Fault, stretching from the western margin of the Ghab Basin (Syria) to the Amik Basin (Türkiye).

The on-land DSFZ has not experienced large-magnitude earthquakes during the instrumental period, but historical records and archaeological data show the occurrence of large-magnitude earthquakes on the DSFZ (Fig. 1). Unless there are historical records with eyewitnesses, the only way to definitively associate historical surface rupture to specific fault segments is via paleoseismologic investigations. In this study we used only historical earthquakes proven to be associated with specific segments and therefore heavily relied on paleoseismological data. From north to south, the most recent surface rupturing earthquake on the Hacıpaşa Fault was the historical 1408 CE earthquake^7,11^. The most recent surface rupturing earthquake on the N–S trending Apame Fault (Fig. 1), bounding the eastern margin of the Ghab Basin, was the 1157 CE event^18,19^. Further south is the Missyaf segment; this section's last surface rupturing earthquake was the 1170 CE event^18^. The NE-SW trending Lebanese Restraining Bend consists of several sub-parallel faults. Still, the main fault is the Yammouneh Fault, and according to^20^, the last surface rupturing event was in 1063 in the northern section and 1202 CE in the southern section. Further south is the Jordan Valley segment of the DSFZ, which last ruptured during the 1033 CE earthquake^21^. The southernmost segment of the DSFZ on land is the Araba Valley Fault (Fig. 1), and the last surface rupturing event on this section was in 1458 CE^22^. The DSFZ has been experiencing approximately 600 years to almost a millennia-long seismic quiescence between the Gulf of Aqaba and Amik Basin (Fig. 1).

### The southern termination of the February 6, 2023, Mw 7.8 Kahramanmaraş earthquake surface rupture

The southwestern extension of the February 6, 2023, Mw 7.8 Kahramanmaraş earthquake surface rupture follows the western margin of the Karasu Valley towards the Amik Basin, where there is a releasing segment boundary associated with an approximately 10 km left step between the fault extending along the western side of the Karasu Valley and the northernmost segment (Hacıpaşa Fault) of the DSFZ (Fig. 2A,B).

**Figure 5:**
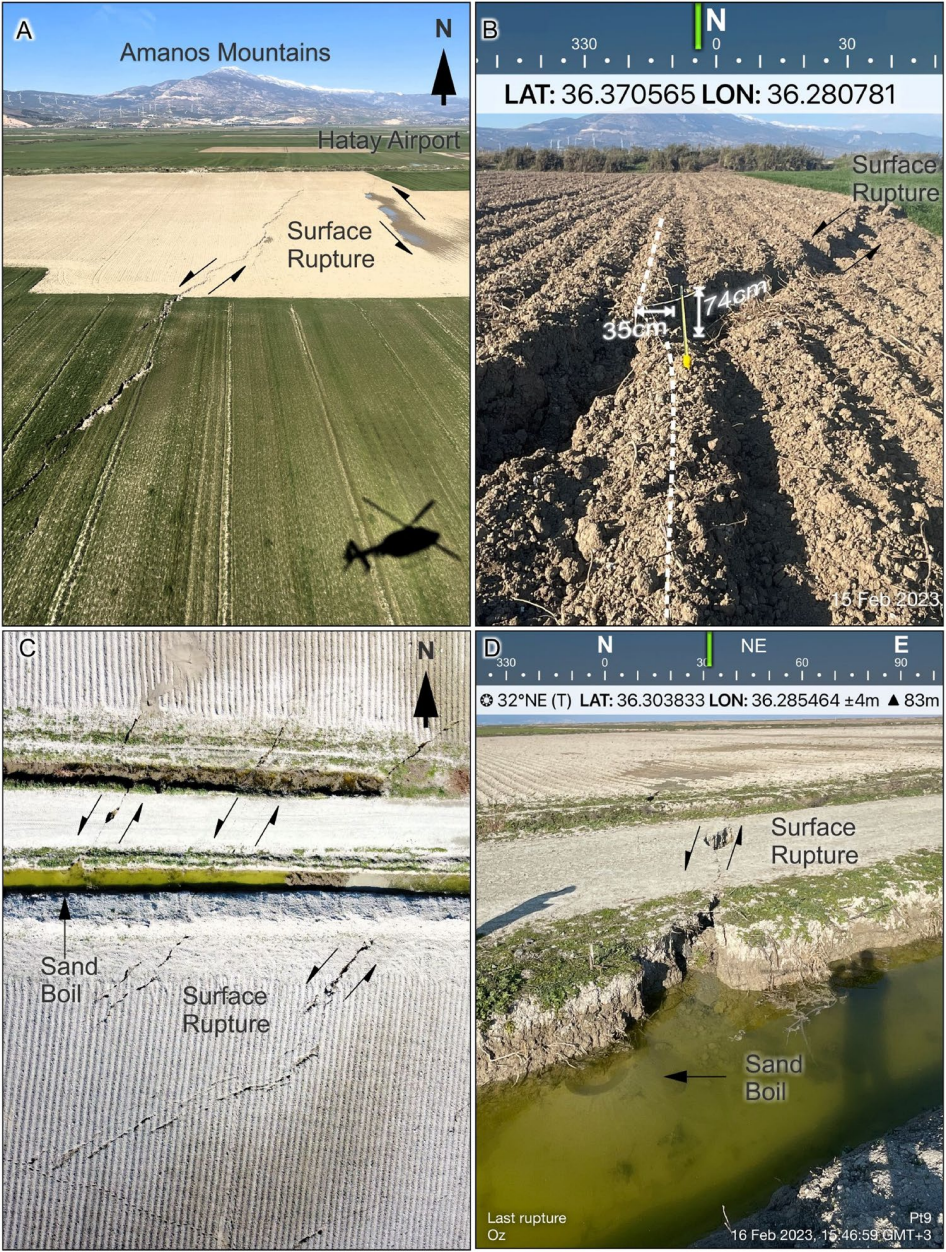
Field observations along the southern surface rupture termination of the M7.8 February 6, 2023 earthquake in the Amik Basin. (**A**) Oblique aerial photograph of the left-stepping surface rupture immediately southwest of the Hatay Airport taken during helicopter reconnaissance. (**B**) Close up view of the surface rupture in a tilled agricultural field approximately 300 m north of the Hatay Airport. (**C**) UAS view of the southern rupture termination near Suvatlı Village approximately 3.5 km south of the Hatay Airport. Note that the surface rupture displays en-echelon cracks; however, it has no measurable offset despite abundant agricultural strain gauges. (**D**) Field photograph of the surface rupture termination as shown in Fig. 5C.

As part of the Geotechnical Extreme Events Reconnaissance, we performed a detailed investigation at the southern end of the Kahramanmaraş earthquake surface rupture^1^. This field reconnaissance within 10 days of the earthquake comprised field observations, unmanned aerial system (UAS) surveys, and a helicopter flight over the Karasu Valley and DSFZ from north of the Hatay Airport to the Syrian border^1^. Our observations show that the southwestern extension of the surface rupture terminated in the Amik Basin (Fig. 2A). Detailed mapping of the southern extent of the surface rupture shows that the left-lateral surface rupture extends in a relatively narrow zone to the town of Kırıkhan (Fig. 2a). From this location southward, it extends with increasing normal and decreasing strike-slip components in a left-stepping en-echelon pattern (Figs. 2B, 5A,B,C). The surface rupture, observed across a ~ 3.5-km-wide deformation zone, terminates immediately west of Suvatlı village (Figs. 2D, 5D, Supplemetary kmz file). The distance from this location to the northernmost segment of the DSFZ is about 7 km, which makes the total width of the releasing step over approximately 10 km (Fig. 2B).

## Discussion and implications

### Possible role of Amik Stepover: inferences from Niksar Stepover and twentieth century NAFZ earthquake sequence

A segment boundary with a certain size and/or level of complexity may act as a barrier to stop the lateral propagation of surface rupture during an earthquake^23–26^. The southern extent of the February 6, 2023, Mw 7.8 Kahramanmaraş earthquake surface rupture dies out in a left-stepping en-echelon pattern within a ~ 10-km-wide releasing step over in the Amik Basin (Figs. 2A,B and 5). This releasing stepover defines the boundary between the reactivated fault during the February 6, 2023, Mw 7.8 earthquake in the west and the northernmost segment of the DSFZ in the east. Coulomb models were developed to understand the stress changes in the region after the 2023 earthquake. While some Coulomb model publications^27,28^ suggest stress drop on the main trace of the northern DSFZ, others^5,29^ suggest stress increase well exceeding the minimal earthquake triggering threshold of ~ 0.01 MPa^30^. After examining Coulomb model publications, we preferred to adapt the USGS model^5^ as others are either too simplified or do not reflect the faults in the region and field observations as accurately.

The role of the ~ 10-km-wide releasing stepover in the Amik Basin presents an intriguing similarity to the role of Niksar Basin along the NAFZ during the 1939 Ms 7.9 earthquake. The rupture of the 1939 earthquake could not propagate across the 10-km-wide Niksar Basin to extend westward along the main trace of the NAFZ (Fig. 2C,D). Instead, the 1939 rupture propagated along south of the Niksar Basin and terminated around Ezinepazar (Fig. 2C). The maximum slip was reported as 7.5 m approximately 65 km west of Erzincan^4^. Co-seismic horizontal displacement was about 3.5 m immediately east of the Niksar Basin, it decreased to ~ 2.5 m and ~ 1.75 m about 20 km and 40 km west of Niksar Basin, respectively, and died out around Ezinepazar^4^. While the Niksar Basin acted as a rupture propagation barrier, the large magnitude 1939 earthquake loaded stress onto the adjacent western NAFZ segment (Fig. 3A)^2^. Three years later, in 1942, the main trace of the NAFZ bounding the northern margin of the Niksar Basin ruptured (Fig. 2D), and the large magnitude surface rupturing earthquakes successively propagated for nearly 800 km westward, reaching the Marmara Sea within 60 years (Fig. 1). A similar condition was observed between the 1999a Izmit and 1999b Düzce earthquakes on the NAFZ. The eastern end of the 17 August 1999 Mw 7.4 Izmit earthquake rupture terminated in the Gölyaka releasing stepover, which is approximately 7 km wide^31^. Three months later, the 12 November 1999 Mw 7.2 Düzce earthquake ruptured the east of the Gölyaka Basin. Both the westward migrating earthquake sequence that started with the 1939 earthquake on the eastern part of the NAFZ and the 1999 earthquakes are unsurpassed examples showing how wide step overs can act as temporary rupture barriers and subsequently large magnitude earthquakes can trigger neighbouring segments. Similarly, we suggest that the ~ 10-km-wide stepover in the Amik Basin acted as a structural barrier, inhibiting the propagation of the earthquake rupture, similar to the Niksar Basin during the 1939 earthquake on the NAFZ. The 2023 surface rupture could not propagate across that releasing segment boundary in the Amik Basin to extend onto the northernmost segment of the DSFZ but loaded stress onto this seismically quiet segment for the past ~ 600 years^5^ (Fig. 3B).

### Implications for size of the next earthquake

Slip rate estimations on the DSFZ rely on geological, geomorphological, archaeoseismological and geodetic data. However, the slip rate on the DSFZ across various spatiotemporal resolutions converges at approximately 4–5 mm/yr^32^, and according to space-based geodetic data, the transient slip rate is 9–10 mm/yr^33^. Considering the slip rate on the DSFZ and the ~ 6–9 century-long seismic quiescence, it is clear that the potential for M > 7 earthquakes is very high. In this context, the southeastward termination of the February 6, 2023, rupture towards the northernmost reach of the DSFZ (i.e. Hacıpaşa Fault) suggests a failed rupture propagation attempt temporarily arrested by this potentially persistent segment boundary. As^2^ demonstrated on the NAFZ, large magnitude earthquake ruptures load stress on neighbouring faults, which sets up the next large one for failure if enough stress has accumulated. Similarly, February 6, 2023 earthquake loaded stress on the neighbouring Hacıpaşa Fault as modelled by^5^ and has increased its potential to rupture. Both archaeoseismological and paleoseismological data show that the DSFZ has been seismically quiet for about 600 years in the north and over 900 years in the south. A similar fault connection geometry at the western end of the 1939 Ms 7.9 earthquake on the NAFZ and the subsequently triggered successive large-magnitude earthquakes migrating westward within a few decades provides a stark analogy highlighting an increased seismic hazard for the entire DSFZ. Following the 1939 earthquake, the NAFZ experienced subsequent rupture north of the Niksar Basin, leading to seven M > 7 cascading seismic events covering nearly 800 km over six decades (Fig. 1). We anticipate a similar phenomenon along the DSFZ following the 2023 Mw 7.8 earthquake, with potentially large magnitude, surface rupturing earthquake sequence starting in the northernmost reach of the DSFZ and migrating southwards at varying intervals, possibly extending to the Gulf of Aqaba (Fig. 1). The combination of over 600–900 years of seismic strain accumulation along the DSFZ at ~ 5 mm/yr (calculated from geological, geomorphological, and archaeoseismological data) and ~ 10 mm/yr (calculated from geodetic data) indicates that 3—9 m slip may be released during M > 7 earthquakes, which would have dramatic consequences considering ~ 30 million inhabitants in southern Türkiye, Syria, Lebanon, Israel, Jordan and Egypt who live within or alongside the DSFZ.

## Conclusion

The ~ 375 km long surface rupture of February 6, 2023, Mw 7.8 Kahramanmaraş earthquake died out within a ~ 10 km wide releasing stepover between the western boundary fault of the Karasu Valley and northern DSFZ (Hacıpaşa Fault). This relationship resembles a similar situation between the 1939 Erzincan (Ms 7.9) and 1942 (Mw 7.0) Niksar-Erbaa earthquakes, which triggered a 60-year-long earthquake sequence with M > 7 along the 800 km of the North Anatolian Fault Zone. February 6, 2023, the Kahramanmaraş earthquake has a similar potential to trigger a cascading earthquake sequence, considering centuries-long seismic quiescence along the DSFZ. The ~ 10 km wide releasing stepover likely arrested the Mw 7.8 Kahramanmaraş earthquake surface rupture further south. However, it loaded significant stress on the Hacıpaşa Fault, the northernmost branch of the DSFZ. The seismic quiescence of the Hacıpaşa Fault since 1408 CE and the 5–10 mm/yr slip rate of the fault increases the probability of the occurrence of another large magnitude (M > 7) earthquake in the same region. When the Hacıpaşa Fault fails with a large magnitude earthquake, it might also trigger an earthquake sequence that includes neighbouring segments towards the south, which have been in a seismic quiescence for more than 600–900 years. The preponderance of the data unequivocally indicates that an 800-km-long fault zone spanning from the Antakya region in Türkiye through western Syria, Lebanon, Israel, Jordan, and eastern Egypt is under significant seismic hazard, necessitating preparedness for large earthquakes exceeding magnitudes M > 7. This heightened seismic risk underscores the imperative for proactive measures to mitigate potential devastation. Compounded by the fragile socio-economical landscape of the region, the urgency of addressing this issue is paramount.

### Supplementary Information

Supplementary Information 1.

## Data Availability

The data used in the study are available from the corresponding author upon reasonable request.
